# Surgical management of post-transplant bronchial stenoses: a single-center experience

**DOI:** 10.1007/s00595-021-02360-z

**Published:** 2021-08-24

**Authors:** Eleonora Faccioli, Andrea Dell’Amore, Pia Ferrigno, Marco Schiavon, Marco Mammana, Stefano Terzi, Federico Rea

**Affiliations:** Thoracic Surgery and Lung Transplantation Unit, Department of Cardio-Thoracic, Vascular Sciences and Public Health, Padua University Hospital, Via N.Giustiniani 2, 35128 Padua, Italy

**Keywords:** Bronchoplasty, Bronchial stenoses, Lung transplantation, Parenchyma-sparing surgery

## Abstract

**Purpose:**

Bronchial stenoses are challenging complications after lung transplantation and are associated with high rates of morbidity and mortality. We report a series of patients who underwent bronchoplasty or sleeve resection for bronchial stenoses that did not resolve with endoscopic treatment after lung transplantation.

**Methods:**

Between 1995 and 2020, 497 patients underwent lung transplantation at our Institution. 35 patients (7.0%) experienced bronchial stenoses with a median time from transplantation of 3 months. Endoscopic management was effective in 28 cases (5.6%) while 1 patient required re-transplantation. Six patients (1.2%) underwent bronchoplasty or sleeve resection.

**Results:**

The procedures of the six patients who underwent bronchoplasty or sleeve resection were as follows: lower sleeve bilobectomy (*n* = 3), wedge bronchoplasty of the bronchus intermedius (*n* = 1), isolated sleeve resection of the bronchus intermedius (*n* = 1), and isolated sleeve resection of the bronchus intermedius (*n* = 1), associated with a middle lobectomy. All patients were discharged after a median time of 11 days. At a median of 12 months from surgery, two patients remain alive with a preserved pulmonary function. Four patients died after a median time of 56 months from bronchoplasty of causes that were not related to surgery.

**Conclusions:**

Bronchial reconstructions are challenging procedures that can be performed in highly specialized centers. Despite this, they can be considered a good strategy to obtain a definitive resolution of stenosis after lung transplantation.

## Introduction

Despite the improvement in surgical technique, bronchial complications continue to represent a significant and challenging problem after lung transplantation, with incidence rates ranging from 1.6 to 33% [1]. Donor and recipient characteristics, hypoperfusion, right-sided anastomoses, organ preservation, primary graft dysfunction, immunosuppression, infections, acute cellular rejection, ischemic time and anastomotic surgical technique represent the main risk factors for airway complications [2].^.^

The clinical presentations of bronchial complications in lung recipients are varied, including focal infection, necrosis, dehiscence, fistulae and granulation tissue formation, stenosis and malacia [3]. Bronchial stenoses are the most common airways complications after lung transplantation with a reported of 1.6–32% [3] and some known risk factors, including dehiscence, necrosis, bacterial (especially *Actinomyces* and *Pseudomonas Aeruginosa*) and/or fungus (*Aspergillus*) infection.

The traditional treatment of post-transplant stenoses is based on endoscopic strategies, such as balloon dilatation, laser ablation, brachytherapy or stenting, which can be performed as single procedures or in combination, while surgical treatments are required as the last step when conservative attempts fail.

We describe the clinical features, surgical techniques and outcomes in a series of six patients who underwent surgery (bronchoplasty or sleeve resection) to treat recalcitrant post-transplant bronchial stenosis.

## Methods

This study was approved by the Institutional Review Board (IRB) of our center (4539/AO/18).

According to our protocol for lung retrieval, the main bronchi are dissected from nodal tissue and divided at a point one to two rings proximal to the upper lobe orifice; subsequent bronchial anastomoses are performed with minimal peribronchial tissue dissection, using absorbable running suture [4-0 Polydioxanone (PDS®), Ethicon Inc, Sommerville, NJ] in the membranous wall and interrupted stitches in the cartilaginous wall. Occasionally, size discrepancies mandate some degree of telescoping, but we generally prefer to perform end-to-end anastomosis. Bronchial anastomosis is routinely wrapped with donor pericardial tissue.

Surveillance bronchoscopy is performed at 1 and 3 months after transplantation, every 3 months during the first year and then annually. Once an airway complication is identified, patients usually undergo bronchoscopy every 2–3 weeks until the healing is complete, otherwise intervention is required. When possible, we usually manage these complications with conservative/endoscopic techniques. Mechanical dilatation represents the first and most frequent approach; other procedures, including laser ablation, high-dose brachytherapy, endobronchial stenting or a combination of these approaches are also applied. The type and distribution of non-surgical treatments in our experience of post-transplant bronchial stenoses are summarized in Table 1.

**Table 1:** Type and distribution of successful non-surgical treatments in cases of post-transplant stenosis

Type of treatment	Number of patients (%)
Mechanical dilatation	15 (53%)
Stenting	9 (32%)
Laser ablation	8 (28%)
Brachytherapy	8 (28%)
Endoscopic toilette/debridement	2 (7%)
Other	3 (11%)

In six cases the stenosis was not responsive to endoscopic treatment and surgical treatment was performed. The surgical strategy depended on the extent and location of the stenosis. We planned to spare the lung parenchyma as much as possible, adopting a variety of sleeve and bronchoplastic resections on the basis of endoscopic and anatomical features. Prior to surgical evaluation, at our center, patients with bronchial stenoses after lung transplantation underwent standard pulmonary function testing; the operability was usually guided, as in the general population, by the value of the forced expiratory volume in 1 s (FEV1) and the diffusion lung capacity for carbon monoxide (DLCO). As a general rule, patients in whom both values are ≥ 80% can tolerate major lung resection, keeping in mind that, in this peculiar subgroup of patients, a reduction of FEV1 does not necessarily reflect a decline in the lung function as it can be a direct consequence of the bronchial stenosis. For this reason, we usually preferred to also perform a perfusion-ventilation scan to predict whether resection would be tolerated.

To better plan the surgical procedure, rigid bronchoscopy was always performed before surgery. All procedures were performed through a right posterolateral thoracotomy in the fifth intercostal space. Anesthesia was maintained by one-lung ventilation using a left-sided double-lumen endotracheal tube. Triple immunosuppressive therapy was continued after the procedure, including the administration of corticosteroids.

## Results

From 1995 to 2020, 497 patients underwent either unilateral or bilateral lung transplantation (BLTX) at our Institution (Thoracic Surgery and Lung Transplant Unit, Padua University-Hospital, Italy). In our transplant case-history, we reported 35 stenotic complications (7.0%). In 26 cases (5.2%) the strictures involved one side, while in 9 patients (1.8%) bilateral stenoses were observed.

Conservative/endoscopic management was effective for 28 patients (5.6%), and showed good outcomes. After excluding one patient who was unresponsive to endoscopic treatment and who required retransplantation due to severe bilateral bronchial stenosis, a conservative approach failed in six patients (1.2%), and surgical treatment was performed.

**Fig. 1:**
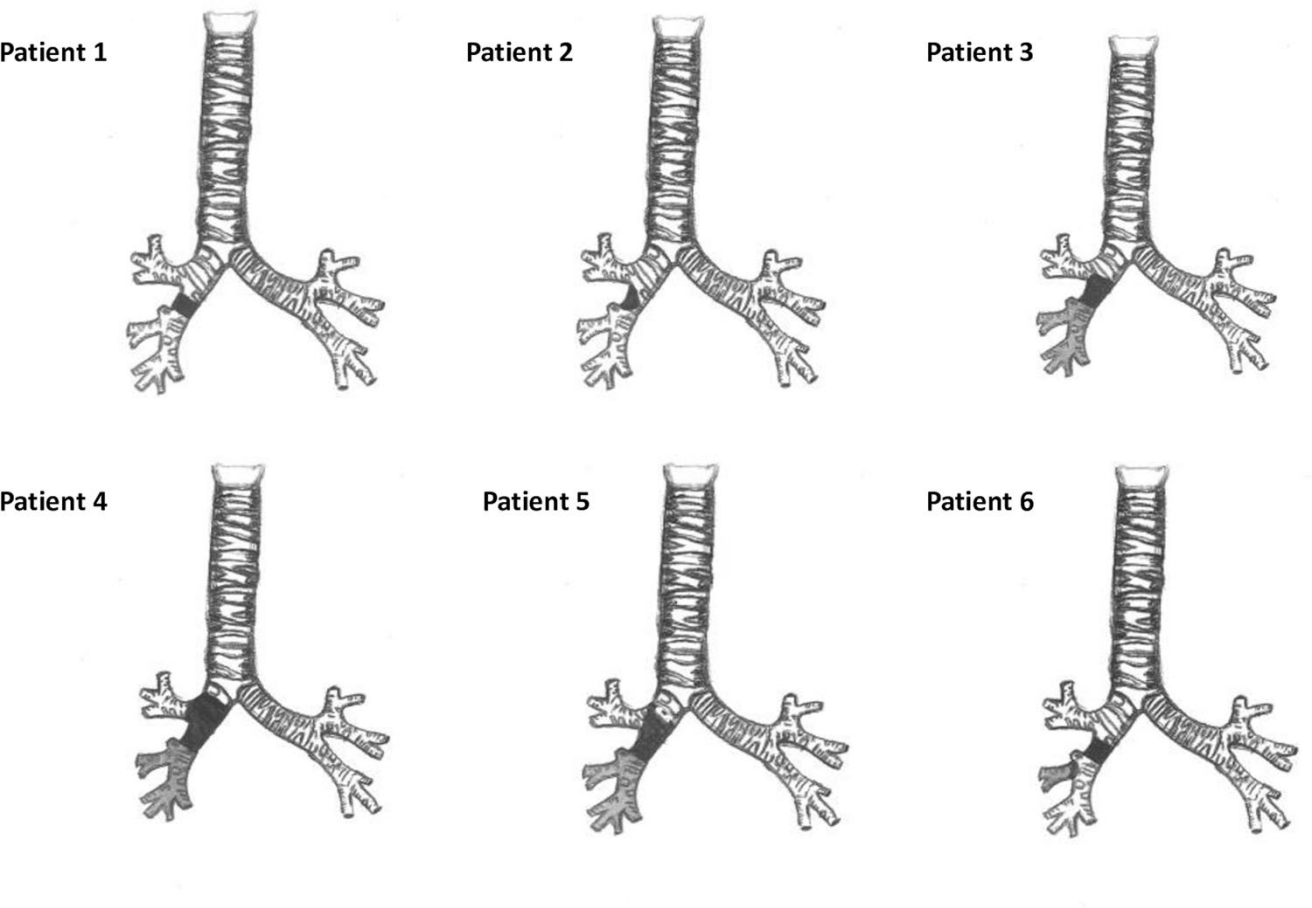
A representative image of the removed stenotic tract (black) and lobectomy performed (grey) for each patient

The main characteristics and perioperative and postoperative outcomes of these six patients are summarized in Tables 2 and 3, respectively. Figure 1 shows the sites of stenosis, the bronchial tract resected and the lobectomy performed for each patient.

**Table 2:** Characteristics of patients who underwent surgical treatment of post-transplant bronchial stenoses

	Sex/age	Disease requiring BLTX	Time between BLTX and stenosis detection (months)	Airway stenosis	Site of bronchial stenosis required management	Endoscopic strategies	Time between stenosis detection and surgical management (months)
Pt 1	M/49 years	Alpha-1 AT deficiency	3	Bilateral	Bronchus intermedius	Endoscopic dilatation, laser ablation, self-expandable stent	1
Pt 2	F/47 years	Bronchiectasis	27	Right–sided	Bronchus intermedius	Endoscopic dilatation, laser ablation	16
Pt 3	M/36 years	CF	3	Right–sided	Bronchus intermedius until the origin of middle and lower right lobar bronchi	Endoscopic dilatation, laser ablation, self-expandable stent	8
Pt 4	M/61 years	COPD	3	Right–sided	Right main bronchus, up to the tracheobronchial angle, bronchus intermedius until the origin of middle and lower right lobar bronchi	Endoscopic dilatation, laser ablation, self-expandable stent	19
Pt 5	F/26 years	CF	11	Right–sided	Bronchus intermedius until the emergence of middle and lower right lobar bronchi	Endoscopic dilatation, laser ablation, brachytherapy	88
Pt 6	M/53 years	Pulmonary fibrosis and Pc-PH secondary to Systemic Scleroderma	2	Right–sided	Bronchus intermedius	Endoscopic dilatation, laser ablation	5

*AT* antitrypsin, *BLTX* bilateral lung transplantation, *CF* cystic fibrosis, *COPD* chronic obstructive pulmonary disease, *F* female, *M* male, *pc-PH* precapillary pulmonary hypertension, *Pt* patient

**Table 3:** Management and post-operative outcomes of surgically treated patients

	Surgical management	MV (hours)	ICU stay (days)	Complications	Hospital days	Status	OS after bronchoplasty (months)	OS after BLTX (months)
Pt 1	Isolated sleeve of the bronchus intermedius	0	0	None	8	Dead—acute pancreatitis	2	7
Pt 2	Isolated bronchoplasty	0	0	None	6	Dead—primary graft dysfunction after retransplantation	89	122
Pt 3	Lower sleeve bilobectomy	0	0	None	10	Dead—massive hemoptysis	97	117
Pt 4	Lower sleeve bilobectomy, anastomosing the tracheo-bronchial angle with upper lobe bronchus	18	2	None	14	Alive	20	43
Pt 5	Lower sleeve bilobectomy anastomosing the tracheo-bronchial angle with upper lobe bronchus	20	8	Airway infection by Pseudomonas aeruginosa	18	Dead—BOS	23	122
Pt 6	Isolated sleeve of the bronchus intermedius and middle lobectomy	0	1	Bronchial fistula	28	Alive	4	12

*BLTX* bilateral lung transplantation, *BOS* bronchiolitis obliterans syndrome, *ICU* intensive care unit, *MV* mechanical ventilation, *OS* overall survival, *Pt* patient

### Patient 1

In November 1996, a 49-year-old man underwent BLTX for alpha-1 antitrypsin deficiency. Three months later, we detected severe stenosis involving the bronchus intermedius, with bronchomalacia and subclinical stenosis of the left upper bronchus. Attempts at endoscopic dilatation, including placement of a self-expandable stent in the stenotic tract, failed. The limited extension of the stenosis allowed the performance of isolated sleeve resection of the bronchus intermedius. After making a right thoracotomy in the fifth intercostal space and identifying the stenosis, the bronchus intermedius was resected upstream and downstream removing the whole stenotic tract. Anastomosis was performed with interrupted 3/0 polyglactin 910 absorbable sutures (Vicryl®, Ethicon, Hamburg, Germany). The total operative time was four hours, and there were no intra-operative complications. The total blood loss was 70 ml. The patient was discharged after 8 days without complications. A pathological examination of the bronchial specimen revealed areas of necrosis caused by cytomegalovirus that could have predisposed the patient to stenosis. Treatment with antiviral agents also allowed progressive resolution of the contralateral stenosis. Endoscopic examinations revealed adequate anastomosis throughout the follow-up period.

In the months that followed, he developed intestinal and pulmonary B-cell lymphoma associated with invasive pulmonary and intestinal aspergillosis. He finally died of hemorrhagic acute pancreatitis, seven months after lung transplantation and two months after the surgical procedure.

### Patient 2

In December 1997, a 47-year-old woman underwent BLTX for bronchiectasis. A mass protruding into the bronchus intermedius was detected during routine bronchoscopy performed 27 months after transplantation. The biopsy specimen was negative for neoplastic cells. Computed tomography (CT) revealed extrinsic compression of the bronchus intermedius in the lower tract, related to the presence of a calcified lymph node simulating granulomatous endoluminal proliferation. Since endoscopic attempts, including balloon dilatation and laser therapy failed, we decided to treat the patient surgically. After performing posterolateral thoracotomy in the fifth intercostal space, the calcified lymph node was found to be strictly adherent to the bronchus intermedius. After careful dissection of the lymph node, which also showed tight adhesion at the esophagus, the bronchus appeared severely stenotic. For this reason, partial resection of the bronchus intermedius wall, followed by isolated bronchoplasty using 4/0 PDS ® continuous suture to reconstruct the defect, was performed. The surgical procedure lasted 6 h with a total blood loss of 120 ml.

The intraoperative and the postoperative courses were uneventful and subsequent bronchoscopic examinations revealed healing at the anastomotic site. At one year after the surgical procedure, partial stenosis due to granulation tissue at the anastomotic site was successfully managed with laser ablation and a temporary placement of an endoprosthesis.

In the years that followed, she developed bronchiolitis obliterans syndrome (BOS), which necessitated unilateral right lung retransplantation at 118 months after the first BLTX. The patient died 85 days after re-transplantation for primary graft dysfunction.

### Patient 3

In June 2005, a 36-year-old man underwent BLTX for cystic fibrosis (CF). Three months later the patient was referred due to persistent cough with purulent sputum that was positive for *Pseudomonas aeruginosa*. Bronchoscopy revealed stenosis involving the bronchus intermedius from its origin to the origin of middle and lower lobar bronchi. During the subsequent years, the stenosis was unsuccessfully managed by endoscopic dilatation and laser ablation. The progressive worsening of the stenosis necessitated the placement of an endobronchial silicone stent in an attempt to maintain airway patency. Endoprosthesis also failed due to the refractory nature of the serrate stenosis. Recurrent episodes of pneumonia led us to perform, through posterolateral thoracotomy, an inferior sleeve bilobectomy with end-to-end anastomosis with triple running 4/0 PDS® sutures between the right main bronchus and the upper lobar bronchus, wrapped with mediastinal fat. No complications were reported during surgery; the total blood loss was 200 ml with an operative time of six and a half hours. The patient’s postoperative clinical course was unremarkable and bronchoscopic examinations performed after 1 and 3 months showed optimal healing of the anastomotic site without bacterial colonization.

In the years that followed, he developed BOS, complicated by recurrent pneumonia due to chronic colonization by *Pseudomonas aeruginosa* until respiratory failure requiring oxygen therapy. He was relisted for re-transplantation, but he died on the waiting list due to massive hemoptysis of unknown cause.

### Patient 4

**Fig. 2:**
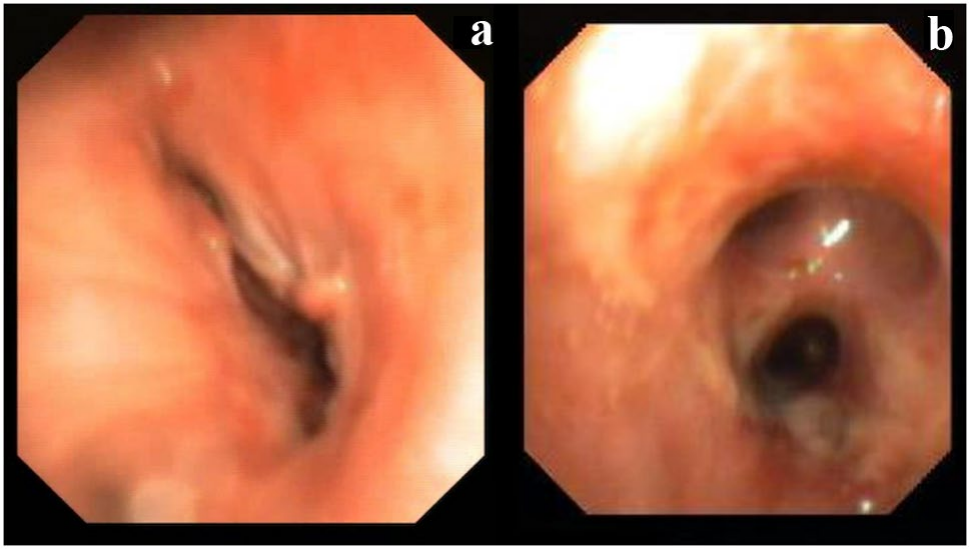
**a** Preoperative bronchoscopy showed evidence of serrate stenosis involving the right main bronchus up to the tracheo-bronchial angle. **b** Postoperative bronchoscopy showed the adequate caliber of anastomosis between the tracheo-bronchial angle and the upper lobar bronchus

In October 2016, a 61-year-old man underwent BLTX for chronic obstructive pulmonary disease. Three months later, during routine bronchoscopy, bronchial stenosis, involving the right main bronchus to the tracheo-bronchial angle (Fig. 2a) and the entire length of the bronchus intermedius, up to the origin of the downstream segmental bronchi, was detected.

Attempts at balloon dilatation and laser therapy failed. A self-expandable stent was also placed in the bronchus but then removed due to the formation of granulomas that worsened the airway stricture.

We finally decided to perform a lower sleeve bilobectomy extended to the right tracheobronchial angle. First, we dissected the bronchus intermedius and then the right tracheo-bronchial angle, with evidence of good distal patency of the upper lobar bronchus. After the removal of the middle and lower lobes, the tracheo-bronchial angle and the upper lobar bronchus were anastomosed with triple running 4/0 PDS® sutures. The anastomotic site was wrapped with perithymic fat and posterior pericardium. No intra-operative complications were reported. The operative time was six hours and the total blood loss was 120 ml.

The clinical course was characterized by persistent air leakage requiring pleural drainage until the tenth postoperative day. Bronchoscopic examinations performed at 1, 3 and 6 months revealed good anastomosis and the caliber of the bronchial lumen was adequate (Fig. 2b). At nine months after the operation, partial stenosis due to granulation tissue was detected. The patient was treated by endoscopic dilatation, which showed good results. At the time of the last follow-up examination, the patient was currently alive, with a good pulmonary function and no evidence of re-stenosis.

### Patient 5

In March 2010, a 26-year-old woman with CF underwent BLTX. Eleven months later, chest X-ray revealed consolidation in the middle and inferior lobes. Bronchoscopy revealed stenosis involving the bronchus intermedius, until the origin of the middle and lower right lobar bronchi. Treatment was attempted several times with mechanical dilatation, laser ablation and brachytherapy. The stenosis of the bronchus intermedius worsened during the subsequent years, resulting in obstructive syndrome with recurrent episodes of pneumonia.

**Fig. 3:**
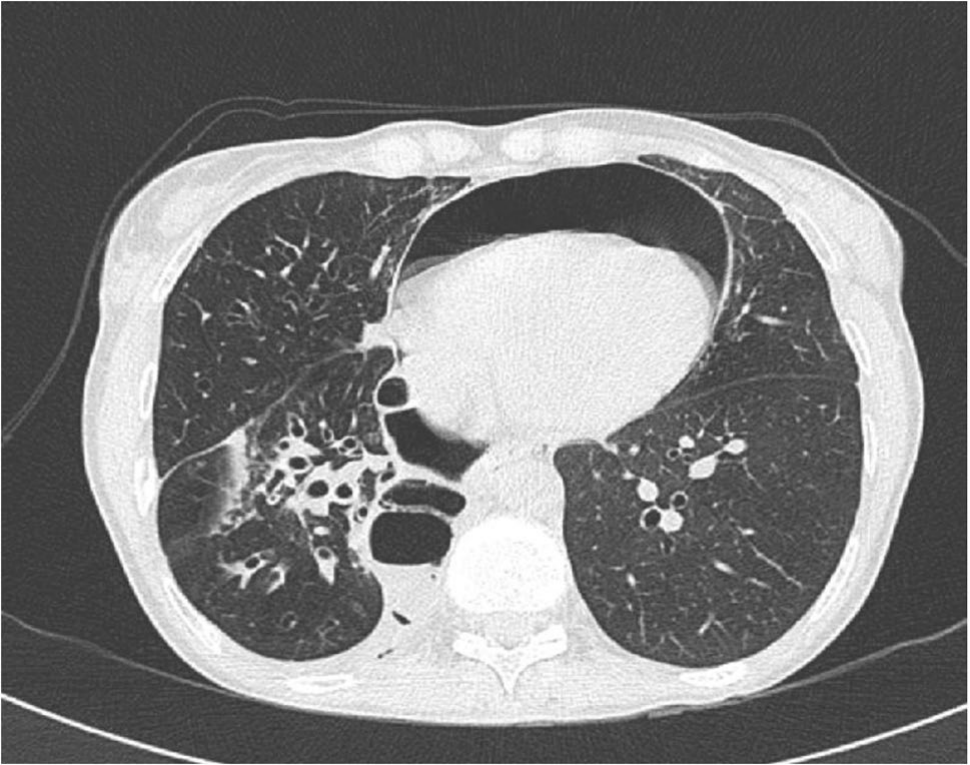
CT scan showing evidence of pneumopericardium consequent to the traumatic rupture of the right bronchial anastomotic line during attempted mechanical dilatation

**Fig. 4:**
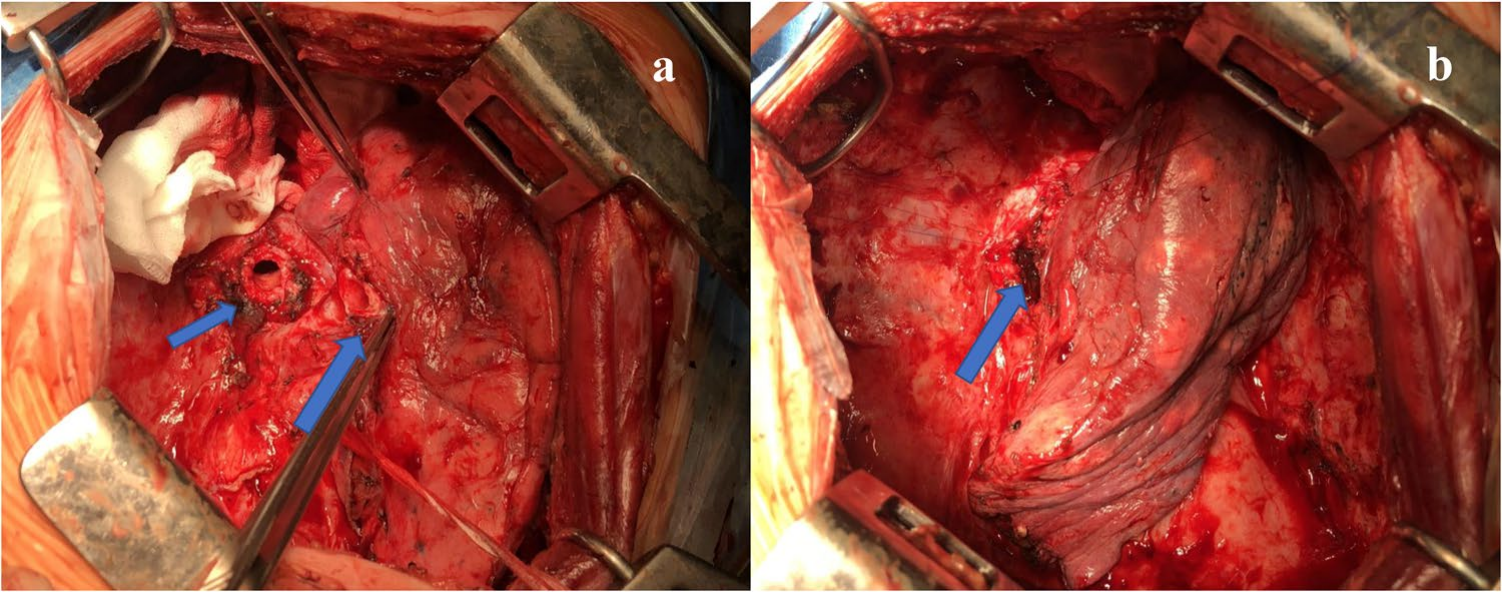
Intra-operative images of the lower sleeve bilobectomy **a** the smallest arrow shows the open right main bronchus while the biggest arrow shows the open right upper bronchus. **b** Anastomosis performed between the right main bronchus and the right upper bronchus

In 2018, during a routine attempt of dilatation by rigid endoscopy, rupture of the right bronchial anastomotic line developed with a consequent pneumopericardium and pneumomediastinum (Fig. 3). Through selective left intubation, the right bronchial hemisystem was promptly excluded and subxiphoid pericardiocentesis was performed, which obtained hemodynamic stability. As the hemodynamic conditions had improved, we decided to perform lower sleeve bilobectomy, performing anastomosis as described in the previous case (Fig. 4). No complications were reported during the surgical procedure, with a total blood loss of 150 ml. The operative time was 2 h and 45 min.

The patient’s postoperative clinical course was complicated by *Pseudomonas aeruginosa* airway infection, which was eradicated after adequate antibiotic therapy. The subsequent endoscopic follow-up showed optimal healing of the anastomotic site. She died 10 years after the first transplant.

### Patient 6

**Fig. 5:**
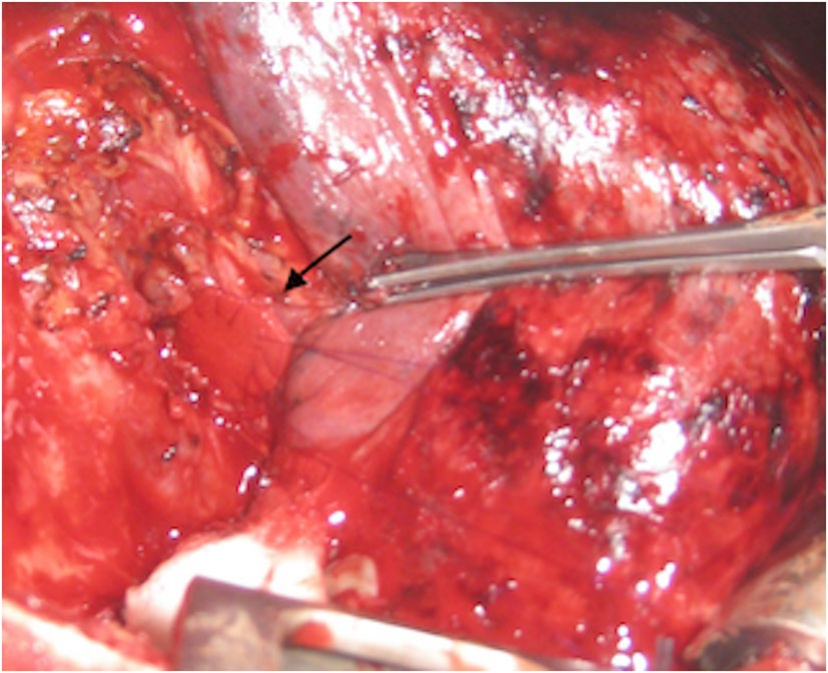
Patch repair of the right perianastomotic line with Gore® Bio-A® tissue reinforcement. Synthetic prosthesis is indicated by black arrow

In May 2019, a 54-year-old man underwent BLTX for pulmonary fibrosis secondary to systemic scleroderma associated with precapillary pulmonary hypertension. At two weeks after BLTX, a chest X-ray revealed bilateral pneumothorax and bronchoscopy showed right perianastomotic dehiscence involving the membranous wall causing a bronchopleural fistula. The gas exchange improved after drainage of the pleural cavity; however, the worsening of the clinical conditions necessitated the surgical repair of the fistula, which was performed by posterolateral right thoracotomy. A prosthetic patch, consisting of Gore® Bio-A® tissue reinforcement (W. L. Gore & Associates, Inc., Flagstaff, AZ), was trimmed and sutured to the membranous wall defect with a continuous 4/0 PDS® suture (Fig. 5) [4]. An intercostal muscle pedicle flap was harvested and applied to the surface of the prosthesis. Finally, surgical tracheostomy was realized. However, the airway manipulation stimulated the proliferation of granulation tissue in the bronchus intermedius, which progressively worsened into a severe stenosis involving the bronchus in its proximal tract.

Bronchoplasty was mandatory due to the failure of attempted endoscopic dilatation. Through a posterolateral re-thoracotomy in the fifth intercostal space, the right lung was entirely mobilized. After extensive dissection of the previous muscle flap wrap, the stenotic area of the bronchus intermedius was identified and resected. Re-anastomosis was performed with running absorbable 5/0 PDS® sutures, realizing an end-to-end anastomosis. Intraoperatively, we observed that the middle lobar bronchus was not patent. This was confirmed by absent ventilation. For this reason, middle lobectomy was performed and the bronchial middle stump was sutured with interrupted 3/0 Vicryl® stitches. There was no significant bleeding during surgery. The total amount blood loss was 100 ml, and the operative time was 4 h.

The post-operative course was complicated by a small bronchial dehiscence at the middle bronchial stump, which was treated conservatively but which required the prolonged pleural drainage. During the follow-up period, endoscopic examinations revealed optimal healing of the anastomotic site, without signs of dehiscence, fistula or stenosis. At the time of writing this report, the patient is alive with a preserved pulmonary function and good quality of life.

## Discussion

Bronchial stenoses, the most frequent post-transplant airway complications, can be located at the bronchial anastomosis or within 2 cm of the anastomotic site (central airways stenoses) otherwise they can involve the airways distal to the anastomotic site (distal airways stenoses). This latter type is less frequent, with a reported incidence of approximately 3%, and is usually located at the bronchus intermedius. This also leads to vanishing bronchus intermedius syndrome, which is found in approximately 2% of cases as late as 12 months from transplantation [5], and which has a significant impact on survival. Despite this and the consideration that the exact pathogenesis of this type of stenosis is not known, we are aware that the bronchus intermedius is especially prone to ischemia after lung transplantation. In our case series the stenosis occurred at this site in all six patients; a possible explanation of this finding may be the refractory nature of this stenosis to endoscopic treatment, which requires surgical treatment to achieve a definitive resolution. Our findings are in line with a French study [6], which reported a high incidence of post-transplant bronchus intermedius stenosis (59%) without the involvement of the anastomotic site, confirming the difficulty in finding a definitive endoscopic treatment for stenoses located at this site.

As a general rule, in the case of post-transplant stenoses, bronchoscopic balloon dilatation is usually the first minimally invasive approach, as it is useful to relieve symptoms. When the stenosis recurs, the placement of an endobronchial stent should be considered as a therapeutic option, knowing that it should only be performed for symptomatic patients who require more than two dilatations per month and in whom the improvement of symptoms after dilatation is confirmed. On the other hand, laser-therapy, brachytherapy, and other debridement techniques can be used to treat bronchial stenosis due to granulation tissue: usually laser therapy provides earlier results than brachytherapy.

Surgical strategies are reserved for cases in which endoscopic treatments fail. Depending on the type, location, and extension of the stenosis, and the anatomy, they can consist of anastomotic reconstitution, bronchoplastic reconstruction, and bronchial sleeve resection with or without parenchyma sacrifice until lung re-transplantation.

In our experience, after the failure of endoscopic treatment, the preferred surgical strategy in patients submitted to lung transplantation should provide the greatest preservation of the parenchyma and is achievable with sleeve resection or bronchoplasty. According to our protocol, each patient underwent pre-operative rigid bronchoscopy to define the extent and localization of the stenosis: surgical procedures were considered feasible, especially in cases of distal stenosis of limited length (< 2–3 cm).

In addition, lung transplant recipients represent a very peculiar population, in which surgical treatment, especially if associated with parenchymal resection, can be very challenging due to issues with anesthesiologic management and the high risk of infections and anastomotic complications, mainly due to the immunosuppressant and anti-inflammatory therapy.

Several recent studies have analyzed the outcomes of lung-sparing surgery in case of malignancies involving the tracheo-bronchial tree [5, 7, 8]; however, evidence on the surgical treatment of post-transplant stenosis remains poor. Despite the high prevalence of this condition, there are no randomized controlled trials on different treatment options and all the evidence on this topic has been extracted from small case series or expert opinions. The first paper reporting the surgical treatment of bronchial stenosis after lung transplantation was published in 1994 [9] by the Hannover Group: in a series of 78 post-transplant bronchial complications, re-intervention was required in 8 patients (10%), among these, surgical treatment was performed in five cases (6.4%) (bilobectomy, *n* = 1; sleeve resection, *n* = 2; re-transplantation, *n* = 2). The three patients who underwent pulmonary resections were discharged after a mean of 23 days and were all alive after a median follow-up period of 12 months.

Paulson et al. [10] reported a single case of non-anastomotic bronchial stenosis in which they performed a parenchymal-sparing sleeve resection of the bronchus intermedius in a 53-year-old patient who underwent bilateral lung transplantation for an alpha1-antitrypsin deficiency.

In 2007, Marulli et al. [11] reported the outcomes of 3 patients with segmental distal stenoses who were successfully treated with surgery after the failure of repeated endoscopic treatment. The following year, Camargo et al. [12] described five cases of bronchial post-transplant complications (stenosis, *n* = 4; broncho-arterial fistula, *n* = 1) that were surgically treated. They performed left upper sleeve lobectomy (*n* = 1), right upper sleeve lobectomy (*n* = 1), and segmental bronchial resection (*n* = 3) with anastomosis, and reported good short- and long-term outcomes with a median hospital stay of 25 days and a median survival period of 32 months. No complications or deaths related to surgical procedures were described.

The outcomes of the published studies on this topic are reported in Table 4. In our case series, which is the largest in the current literature, we reported a median hospital stay of 11 days, which was only higher than the single case reported by Paulson [10]; however, it should be noted that his patient underwent isolated sleeve resection without any resection of the parenchyma. On the other hand, the Hannover group [9] had a sensibly higher duration of hospital stay (mean hospital stay: 42 days), but it can be justified if we consider that they also reported two cases of re-transplantation in their case series.

**Table 4:** Outcomes of the main studies in current literature

Author	Year	No of patients	Type of airway’s complication (n)	Type of surgical treatment	Early post-operative complications	Median hospital stay (days)	Median follow-up (months)
Schafers [9]	1994	5	Bronchial stenosis (4) Broncho-arterial fistula (1)	Bilobectomy (1) Sleeve resection (2) Re-transplantation (2)	No	42 (mean)	12
Paulson [10]	2003	1	Bronchial stenosis	Sleeve resection	No	6	6
Marulli [11]	2007	3	Bronchial stenosis	Lower sleeve bilobectomy (1) Wedge bronchoplasty (1) Isolated sleeve resection (1)	No	NR	NR
Camargo [12]	2008	5	Bronchial stenosis (4) Broncho-arterial fistula (1)	Sleeve lobectomy (2) Segmental bronchial resection (3)	No	25	32

*N* number, *NR* not reported

Concerning the long-term outcomes, our median follow-up period was 20 months, which was only inferior to that reported by the Brazilian group [12]; the explanation is that three of our cases underwent surgical treatment in the last two years; thus, the duration of follow-up was reduced. Furthermore, we had no deaths or major complications related to surgical treatments (with the exception of dehiscence, for which conservative treatment was successful) in the post-operative period; this finding is in line with the studies reported in Table 4.

In conclusion, in this high-risk population, the decision to perform surgery—which includes various bronchoplasty or bronchial sleeve resection procedures—depends on different factors, including anatomical characteristics, the extension of the stenosis, failure of previous endoscopic treatments, clinical conditions and center expertise. These techniques represent a challenging procedure that needs to be meticulously performed and which can only be realized in highly specialized centers. Despite all of these factors, we think that bronchoplasty with or without resection of the parenchyma can be a good strategy to obtain definitive resolution of bronchial stenosis after lung transplantation.
